# Propofol elicits autophagy via endoplasmic reticulum stress and calcium exchange in C2C12 myoblast cell line

**DOI:** 10.1371/journal.pone.0197934

**Published:** 2018-05-24

**Authors:** Xi Chen, Long-Yun Li, Jin-Lan Jiang, Kai Li, Zhen-Bo Su, Fu-Qiang Zhang, Wen-Jing Zhang, Guo-Qing Zhao

**Affiliations:** 1 Department of Anesthesiology, China-Japan Union Hospital of Jilin University, Changchun, China; 2 Department of Research Center, China-Japan Union Hospital of Jilin University, Changchun, China; Univerzitet u Beogradu, SERBIA

## Abstract

In this study, we investigated the relationship between propofol and autophagy and examined whether this relationship depends on ER stress, production of ROS (reactive oxygen species), and disruption of calcium (Ca^2+^) homeostasis. To this end, we measured C2C12 cell apoptosis *in vitro*, along with Ca^2+^ levels; ROS production; and expression of proteins and genes associated with autophagy, Ca^2+^ homeostasis, and ER stress, including LC3 (microtubule-associate protein 1 light chain 3), p62, AMPK (adenosine 5’-monophosphate (AMP)-activated protein kinase), phosphorylated AMPK, mTOR (the mammalian target of rapamycin), phosphorylated mTOR, CHOP (C/BEP homologous protein), and Grp78/Bip (78 kDa glucose-regulated protein). We found that propofol treatment induced autophagy, ER stress, and Ca^2+^ release. The ratio of phosphorylated AMPK to AMPK increased, whereas the ratio of phosphorylated mTOR to mTOR decreased. Collectively, the data suggested that propofol induced autophagy *in vitro* through ER stress, resulting in elevated ROS and Ca^2+^. Additionally, co-administration of an ER stress inhibitor blunted the effect of propofol.

## Introduction

Propofol (2,6-diisopropylphenol) is an intravenous anesthetic widely used to induce and maintain anesthesia during surgery, as well as to sedate patients with critical illnesses such as sepsis [1]. Propofol activates GABA_A_ receptors and inhibits NMDA receptors [2]. It significantly increases peak and integrated calcium (Ca^2+^) responses [3]. Accordingly, propofol protects animals against septic shock [4, 5], ischemia-reperfusion injuries [6, 7], and oxidative stress [8, 9]. Finally, a growing body of evidence suggests that propofol regulates immunity by suppressing NO biosynthesis in LPS-activated macrophages [10], by attenuating LPS-stimulated cytokine production in cultured hepatocytes [11], or by preventing endotoxemia-induced and hyperglycemia-induced dysfunction in endothelial cell barriers [12, 13]. Moreover, propofol contains a phenolic hydroxyl group similar to that of vitamin E and exhibits antioxidant activity by scavenging free radicals [14].

Propofol protects rat cardiomyocytes and hippocampal neurons [6] against ischemia/reperfusion-induced autophagic cell death. Autophagy is an important regulatory mechanism, and its inhibition may either result in direct cell death or sensitize cells to stimuli-induced damage [15, 16]. Recent advances suggest a possible link between autophagy and anesthetic-induced cytotoxicity. For example, Morissette *et al* [17] reported that smooth muscle cell death due to the local anesthetics bupivacaine and lidocaine was associated with increased autophagy. Additionally, others have demonstrated *in vitro* that autophagosome accumulated in cells exposed to the local anesthetic dibucaine [18].

However, the effects of general anesthetics on autophagy are unclear. Additionally, the results from experiments testing these effects require careful consideration, as the effects may be masked or confounded by the associated therapeutic interventions. For instance, although general anesthesia is already known to boost autophagy in some organs [19], the time course of this effect and its underlying mechanism are unknown, especially in skeletal muscles. In addition, muscle atrophy due to prolonged sedation or general anesthesia may also be a result of increased autophagy [20]. The endoplasmic reticulum (ER) is an organelle that regulates protein secretion, cell surface development, and the Ca^2+^ concentration in cells [21]. Regions of the ER membrane enriched with Ca^2+^-binding chaperones that interact with mitochondria are called mitochondria-associated ER membranes (MAMs), which preserve and regulate cellular homeostasis when cells are exposed to different stimuli [22]. ER stress is linked closely to changes in the composition of MAMs, deregulation of Ca^2+^ transport, and induced cell death [23]. In this study, we evaluated whether the effects of propofol on autophagy, if any, were associated with ER stress and Ca^2+^ homeostasis.

## Results

### Dual effects of propofol on C2C12 cells

**Fig 1:**
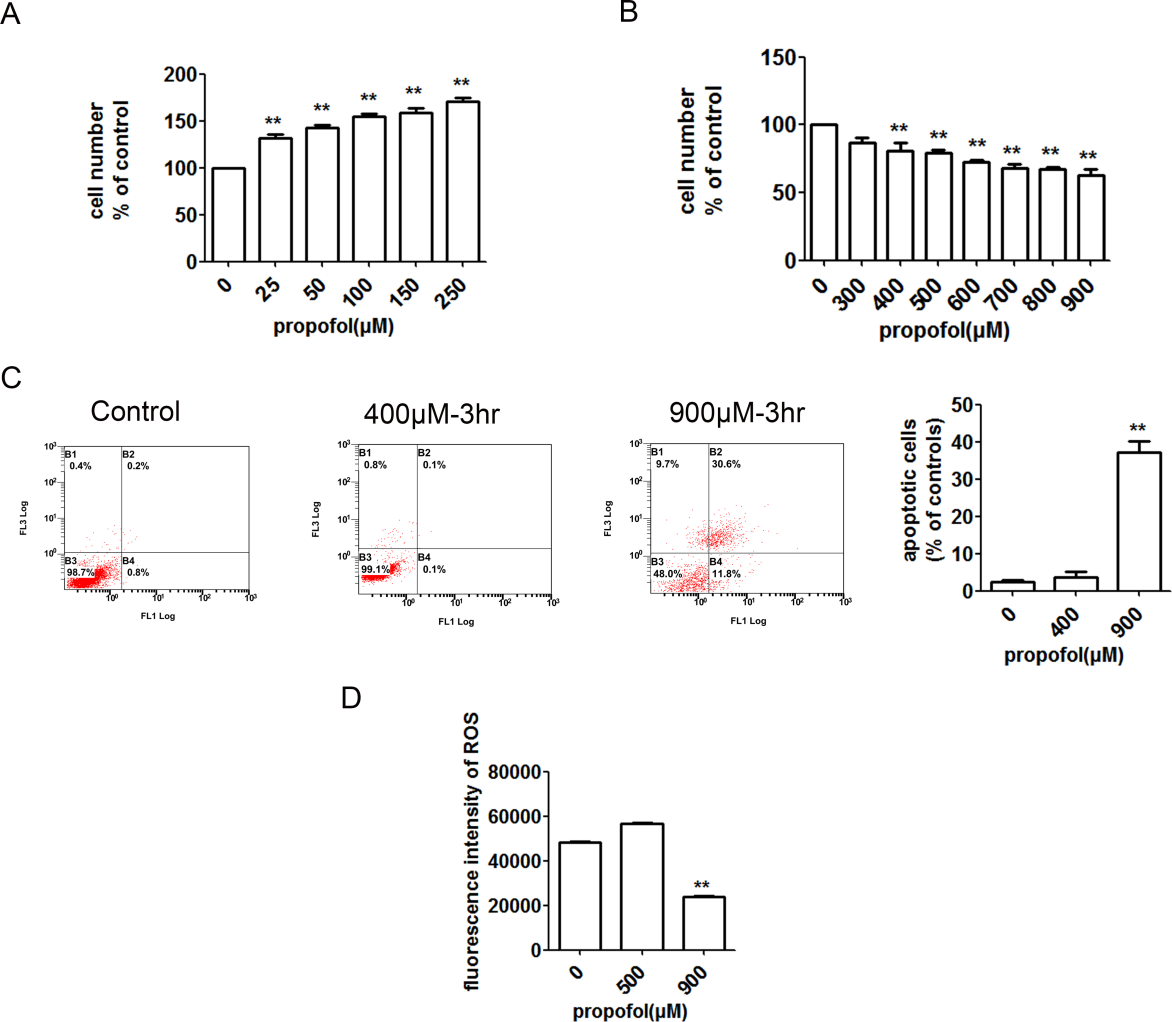
Dual effects of propofol on C2C12 cells. (A) Exposure for 48 h to 25, 50, 100, 150, and 250 μM propofol significantly increased cell count relative to that of the control in a concentration-dependent manner. Each bar represents a mean ± standard deviation (SD); *, P < 0.05; **, P < 0.01, n = 3 per group. (B) Compared to the control, viability was lower in cells incubated for 48 h with 300, 400, 500, 600, 700, 800, and 900 μM propofol. Each bar represents the mean ± SD; *, P < 0.05 vs untreated controls, **, P < 0.01; n = 3 per group. Cell viability was measured by CCK-8 assay. (C) Apoptosis was analyzed and quantified by annexin V/PI staining and flow cytometry. FL1 represents FITC and FL3 represents PI. Data are presented as mean ± SD; **, P < 0.01; n = 3 per group. (D) Production of ROS, as measured by green fluorescence of 2,7-dichlorodihydrofluorescein diacetate (H2-DCFDA). ROS increased significantly in cells treated with 400 μM propofol for 3 h, but decreased significantly in cells treated with 900 μM, as measured by FlowJo. Each bar represents a mean ± SD; **, P < 0.01; n = 3 per group.

General anesthesia is associated with metabolic changes in various organs, including both upregulation and suppression of apoptosis [24–27]. In addition, general anesthetics typically protect many organs [28], but are neurotoxic in some cases [29]. We previously determined safe, non-toxic doses of propofol to treat cells. Thus, it seems important to examine the effect of anesthesia on C2C12, since it is associated with both apoptosis and autophagy. As shown in Fig 1A, exposure for 48 h to 50, 150, and 250 μM propofol promoted cell viability in a concentration-dependent manner. However, higher concentrations of propofol reduced cell viability relative to that of control, as shown in Fig 1B. Furthermore, we found that apoptosis was induced after 3 h at 900 μM but not at 400 μM (Fig 1C), indicating that the latter dose is safe and nontoxic; this dose was used in all subsequent experiments unless stated otherwise. Notably, we found that ROS increased at 400 μM, but decreased at 900 μM (Fig 1D), suggesting that propofol selectively induced ROS production and autophagy, but not apoptosis. How propofol induces apoptosis requires further research and is beyond the scope of this work, which focuses on whether propofol induces autophagy and through what mechanism.

### Autophagy is induced in propofol-treated C2C12 cells in a concentration-dependent manner

**Fig 2:**
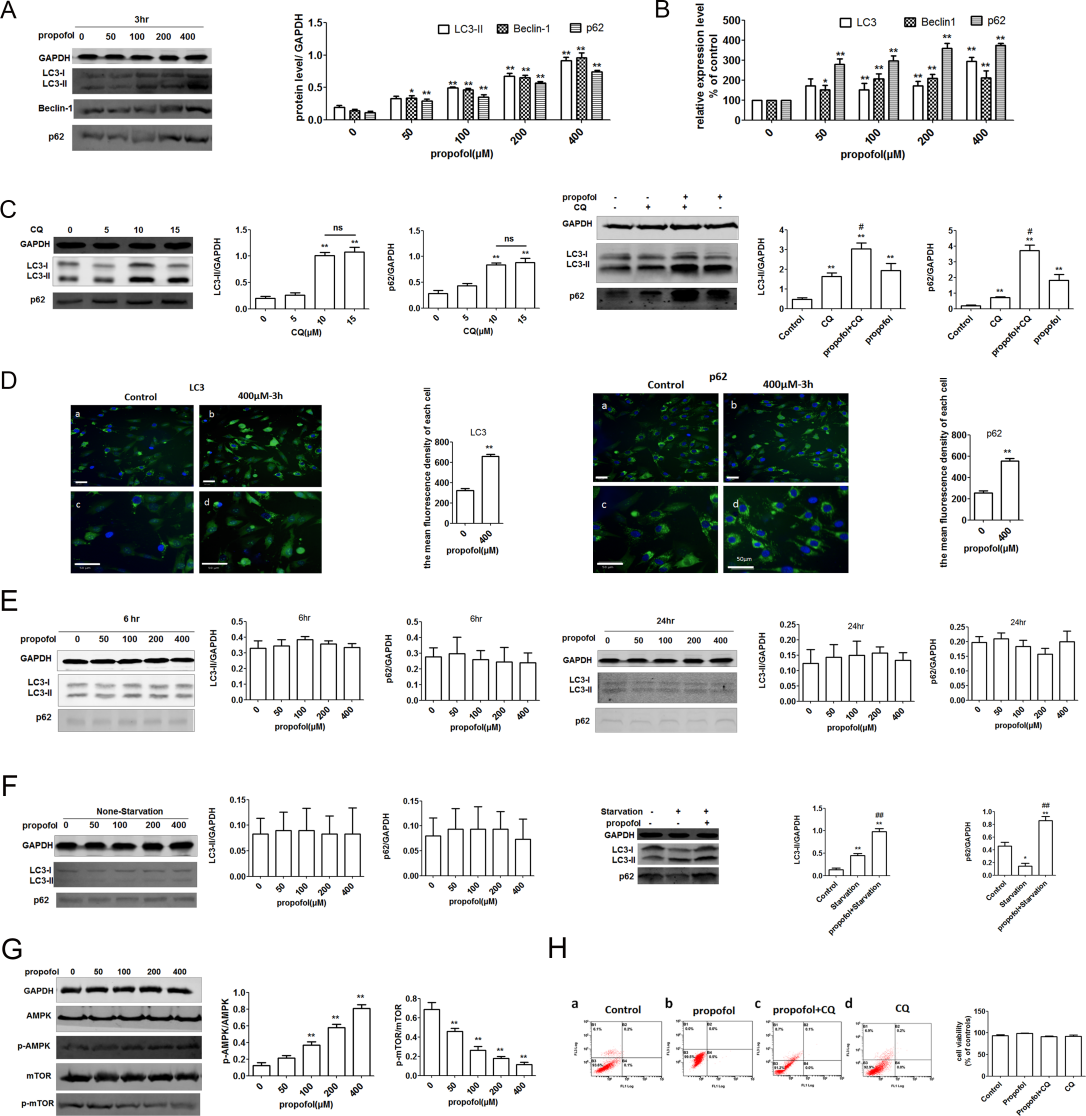
Autophagy is induced in propofol-treated C2C12 cells in a concentration-dependent manner. (A) LC3-II, p62 and Beclin-1 were upregulated in C2C12 cells treated with 50, 100, 200, and 400 μM propofol for 3 h, as measured by western blot. (B) In C2C12 cells treated with 50, 100, 200, and 400 μM propofol for 3 h, p62 and Beclin-1 gene (*BCN1*) expression increased with propofol concentration, as measured by qRT-PCR. LC3 gene expression also increased, but independently of propofol concentration. Each bar represents a mean ± SD; n = 3; *, P < 0.05 vs untreated controls. (C) LC3-II and p62 were tested in C2C12 cells treated with 0, 5, 10 and 15 μM propofol for 3 h, as measured by western blot. C2C12 cells were treated with 400 μM propofol for 3 h with or without chloroquine (CQ). Expression of LC3 and p62 were detected by western blot analysis. Each bar represents a mean ± SD; *, P < 0.05; **, P < 0.01 vs untreated controls; n = 3 per group; #, P < 0.05 (comparison with cells treated with propofol) (D) Immunofluorescence analysis of LC3 and p62 expression in C2C12 cells treated with or without Propofol (400 μM) for 3 h. Nuclei were stained with Hoechst (scale bar, 50 μM). Fluorescence intensity was calculated and determined for each cell. Each bar represents a mean ± SD; *, P < 0.05; **, P < 0.01; comparison with untreated controls, n = 3 per group. (E) LC3 and p62 were hardly detected in C2C12 cells treated with 50, 100, 200, and 400 μM propofol for 6 h and 24 h, as measured by western blot. (F) Detection of indicated autophagy-essential protein LC3 and p62 by western blot in C2C12 cells with starvation and propofol treatment. LC3 and p62 were not altered in none-starved cells treated with 50, 100, 200, and 400 μM propofol for 3 h, as measured by western blotting. *, P < 0.05; **, P < 0.01 vs untreated controls; n = 3 per group; ##, P < 0.01 (comparison with starvation treated) (G) C2C12 cells treated with 50, 100, 200, and 400 μM propofol for 3 h were analyzed by western blot for phosphorylated AMPK and mTOR. (H) Apoptosis was quantified by flow cytometry. FL1 represents FITC and FL3 represents PI. (a) control; (b) Propofol (400 μM); (c) Propofol (400 μM) + CQ (10 μM); (d) CQ (10 μM). Each bar represents a mean ± SD; *, P < 0.05; **, P < 0.01 comparison with controls; n = 3 per group.

The expression of Beclin-1 as an indicator of autophagy induction [30, 31], was measured by western blot and qRT-PCR in C2C12 cells exposed to propofol. As the level of LC3-II correlates with the number of autophagosomes, this characteristic conversion of LC3 can be used to monitor autophagic flux. LC3-II and Beclin-1 were upregulated in a concentration-dependent manner (Fig 2A). Similar trends were observed for their relative gene expression (Fig 2B). Moreover, the level of p62 has been assayed as a measure of substrate sequestration into autophagosome, and it has been reported that it binds LC3 and substrates marked for degradation [17, 32, 33]. Similar to Beclin-1 and LC3B-II, p62 accumulates in a concentration-dependent manner (Fig 2A). To clarify this point, a flux experiment was performed. We examined the ability of propofol to promote autophagy in C2C12 cells, in the presence or absence of CQ, which is a weak basic amine that inhibits the late stage of autophagy by increasing the lysosomal pH and inhibiting lysosomal enzymes post-sequestration. As shown in Fig 2C, exposure for 3 h to 0, 5 and 10 μM CQ promoted LC3-II and p62 increasing in a concentration-dependent manner. However, no further effects were observed when C2C12 cells were treated with 15 μM CQ. These results suggested that 10 μM CQ was safe and optimal to cause a maximum increase in LC3-II and p62 expression. The level of LC3-II expression induced by propofol when in association with CQ was significantly higher than that induced by propofol or CQ alone (Fig 2C). This indicated that autophagy was, indeed, activated. The possibility of reduced autophagosome clearance occurring in the present experimental setting cannot be discarded. A genetic experiment revealed that p62 transcript level increased (Fig 2B), suggesting that p62 accumulation may result from transcriptional induction and, possibly, reduced degradation. Simultaneously, immunofluorescence analysis of cells revealed that in cells exposed to 400 μM propofol, LC3 and p62 were localized to punctate cytoplasmic structures (Fig 2D). Images were taken using the high-throughput optical imager Operetta, and the level of fluorescence was calculated using the integrated Columbus image data storage and analysis system to obtain the mean fluorescence intensity of each cell. LC3 and p62 protein were hardly detectable in cells exposed to propofol for 6 h or 24 h (Fig 2E).

To confirm whether the anesthesia itself and not starvation treatment causes autophagy, distinctions were made between state of anesthesia and state of starvation. As shown in Fig 2F both starvation and propofol induced autophagy. Compared with starvation, propofol induced more amount of autophagy for the same period (Fig 2F). These results suggested that anesthesia-induced autophagy is caused by propofol itself and possibly starvation. Afterward, to confirm the result, we examined the effect of propofol on autophagy in non-starved cells. As shown in Fig 2F, expression of LC3-II and p62 were not altered by propofol treatment. Our results suggested that propofol-induced up-regulation of autophagy was at least partially related to starvation.

We investigated potential molecular mechanisms driving propofol-induced autophagy in C2C12 cells. Western blot analysis suggested that propofol inhibited the phosphorylation and activation of mammalian target of rapamycin (mTOR), an important regulator of autophagy. In addition, we found that phosphorylated AMP-activated kinase (AMPK), an inhibitor of mTOR, was upregulated in propofol-treated cells (Fig 2G). These data suggest that autophagy is activated in propofol-treated cells. We evaluated cell viability when cells were treated with CQ, propofol, or both to further investigate the role of autophagy in propofol-promoted cell viability. As shown in Fig 2H, apoptotic cells treated with propofol were hardly detected regardless of whether CQ treatment was applied.

### ER stress and calcium homeostasis played key roles in propofol-induced autophagy

**Fig 3:**
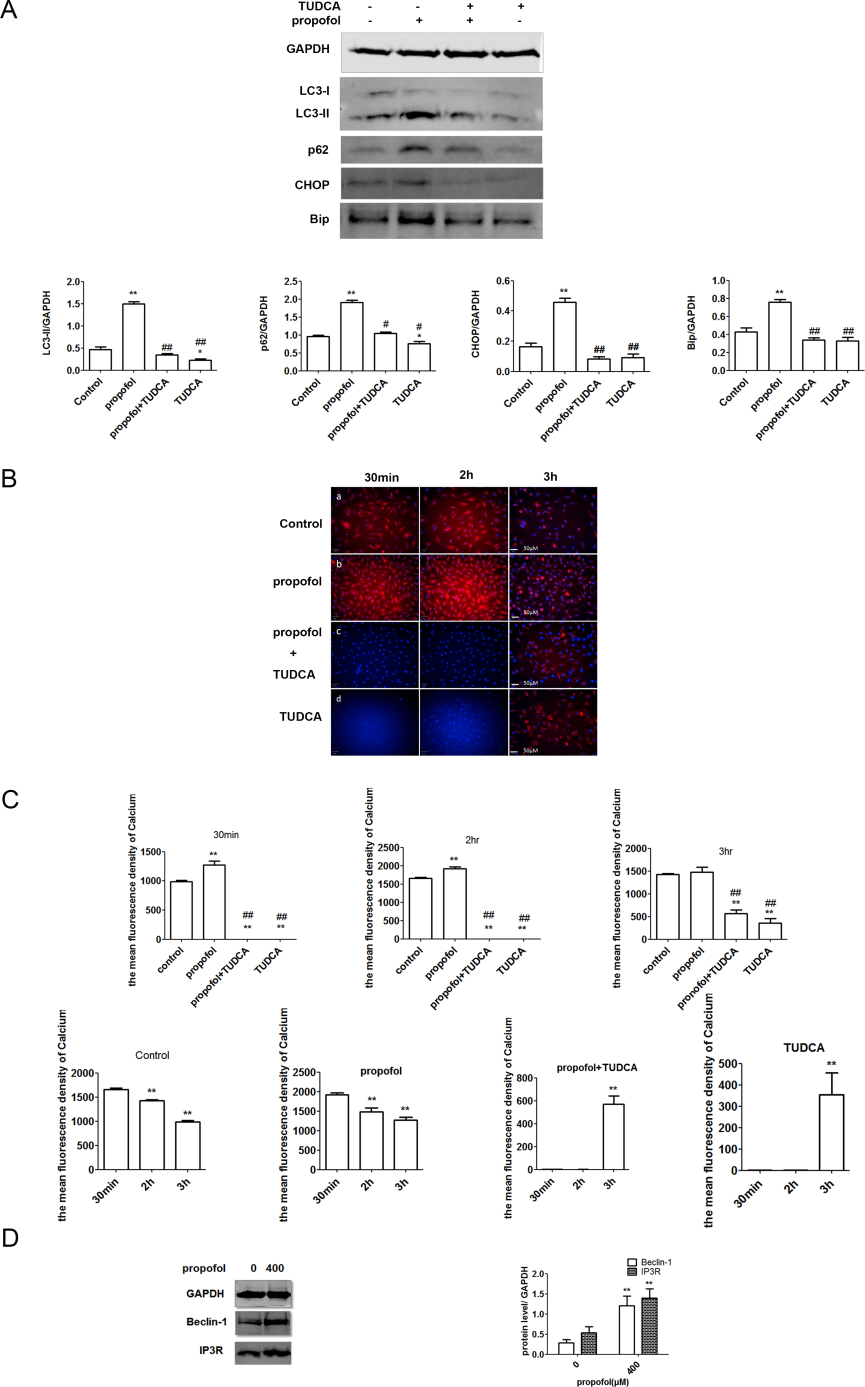
ER stress and calcium homeostasis played key roles in propofol-induced autophagy. (A) C2C12 cells treated with propofol (400 μM) for 3 h with or without TUDCA (1 mM). CHOP, Bip, LC3, and p62 levels were detected by western blot analysis. The LC3-II/GAPDH and p62/GAPDH ratios increased only in cells exposed to propofol. Each bar represents a mean ± SD; n = 3; *, P < 0.05 vs. untreated controls; **, P < 0.01; ^##^, p < 0.01 (comparison with cells treated with propofol). (B) C2C12 cells treated with propofol (400 μM), TUDCA (1 mM), or both. Ca^2+^ was stained by Rhod-2 AM and red fluorescence was induced in control cells and cells treated with propofol for 30 min, 2 h, and 3 h (scale bar, 50 μM). (C) To detect the concentration of Ca^2+^, Ca^2+^ was stained by Rhod-2 AM and red fluorescence was induced. The y-axis represents the mean intensity of red fluorescence in each cell; each bar represents a mean ± SD; n = 4; *, P < 0.05 vs untreated controls; ^##^, p < 0.01 (comparison with cells treated with propofol). (D) IP3R and Beclin-1 expression levels were measured by western blot. CHOP, C/BEP homologous protein; Bip/Grp78, 78 kDa glucose-regulated protein; IP3R, inositol triphosphate receptor.

Skeletal muscle remodeling in response to muscle disuse is known to be associated with ER stress, which, in turn, stimulates autophagy and contributes to muscle atrophy. Indeed, autophagy due to ER stress is possibly a molecular mechanism by which muscle homeostasis is maintained in response to disuse and unloading. Therefore, we investigated autophagy in C2C12 cells exposed to propofol and TUDCA, a well-known inhibitor of ER stress [34]. Treatment with TUDCA in the presence or absence of propofol reduced expression of ER stress markers, as measured by western blot (Fig 3A). In particular, expression of LC3-II, Bip, and CHOP increased upon exposure to propofol, but did not increase in the presence of TUDCA. The LC3-II/GAPDH and p62/GAPDH ratios were also determined. The LC3-II/GAPDH ratio increased when cells were treated with propofol. These results showed that propofol treatment increases ER stress in C2C12 cells.

Disruption of the homeostasis between cytosolic and organellar Ca^2+^ may affect autophagy and a number of other Ca^2+^-dependent cellular processes. Ca^2+^ is also considered to be a secondary messenger strongly involved in the ER stress response [35]. Hence, propofol may also impact Ca^2+^-dependent signaling cascades. To quantify changes in cellular Ca^2+^, cells exposed to propofol with or without TUDCA were stained with Rhod-2AM, an indicator of intracellular Ca^2+^, and imaged using an Operetta high-throughput optical imager (Fig 3B). Ca^2+^ levels were calculated using the integrated Columbus image data storage and analysis system, and the mean fluorescence intensity of Ca^2+^ in each cell is shown in Fig 3C. We compared the fluorescence of the four different groups at several time points. In the first 30 min, fluorescence intensity increased more in the cells treated with propofol than in the control group. In the first 2 h, Ca^2+^ homeostasis was disrupted by propofol, whereas fluorescence was hardly detected in cells exposed to TUDCA with or without propofol. In addition, we analyzed fluorescence within groups at different times. The mean fluorescence intensity increased in the first 30 min after exposure to 400 μM propofol, but then decreased in the next 2 h. The mean fluorescence intensity decreased significantly after exposure for 3 h. A similar trend was observed in the control group. In cells exposed to TUDCA with or without propofol, fluorescence was undetectable in the first 2 h before increasing by 3 h. Collectively, these results indicated that propofol disrupted Ca^2+^ homeostasis and increased cytoplasmic Ca^2+^.

IP3R-mediated Ca^2+^ release from the ER is associated closely with increased levels of free cytoplasmic Ca^2+^, which activates autophagy [36]. IP3R controls Ca^2+^ exchange between the ER and the mitochondria, typically through interactions with Beclin-1 and Bcl-2. Indeed, Beclin-1 co-immunoprecipitates with IP3R in a Bcl-2-dependent manner. Thus, we measured Beclin-1 and IP3R expression by western blot following propofol treatment. We found that the expression increased in C2C12 cells treated with 400 μM propofol along with that of IP3R (Fig 3D). Taken together, the data implied that propofol disrupts Ca^2+^ homeostasis directly by regulating Beclin-1 and its interaction with IP3R, thereby inducing ER stress.

### Propofol induced a burst of ROS

**Fig 4:**
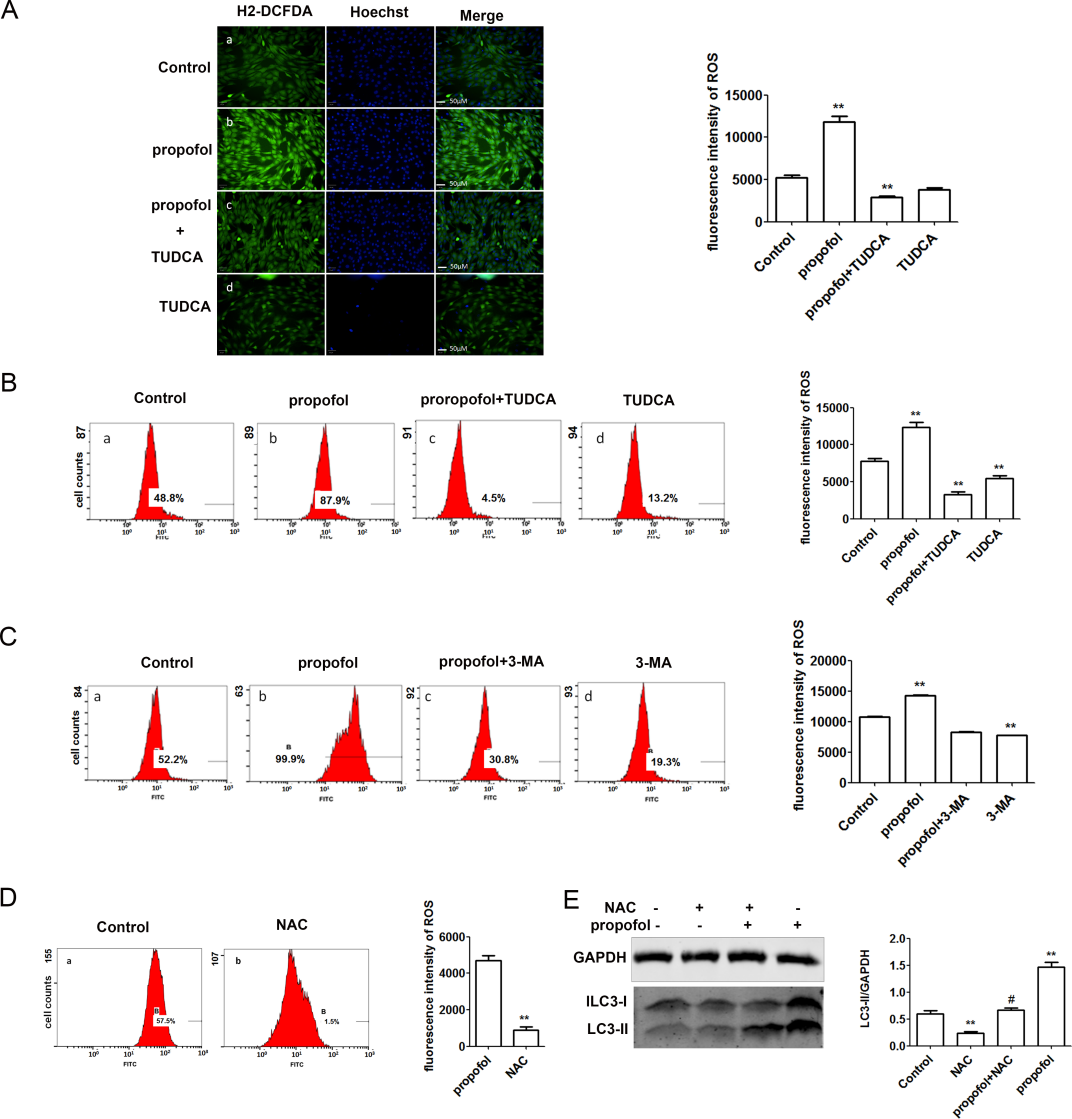
Propofol induced a burst of ROS. (A) Production of ROS, as measured by 2,7-dichlorodihydrofluorescein diacetate (H2-DCFDA) (left panels); nuclei were stained with Hoechst (middle panels). In merged images (right panels), the green fluorescence increased in cells treated with 400 μM propofol, but decreased in cells treated with TUDCA. In the quantification of ROS production measured by H2-DCFDA, the y-axis represents the mean intensity of green fluorescence. Data are presented as mean ± SD; *, P < 0.05; n = 3 per group. (B) Production of ROS, as measured by H2-DCFDA; fluorescence-activated cell sorting (FACS) was performed. ROS increased in cells treated with 400 μM propofol but decreased in cells treated with TUDCA. (a) Control; (b) Propofol (400 μM); (c) Propofol (400 μM) + TUDCA (1 mM); (d) TUDCA (1 mM). Y-axis represents cell counts, x-axis represents intensity of green fluorescence. (C) Production of ROS, as measured by H2-DCFDA; FACS was performed. ROS output also decreased in cells treated with 3-MA, alone or in combination with propofol. (a) Control; (b) Propofol (400 μM); (c) Propofol (400 μM) + 3-MA (10 mM); (d) 3-MA (10 mM). Y-axis represents cell counts, x-axis represents intensity of green fluorescence. Data are presented as mean ± SD; *, P < 0.05; n = 3 per group. (D) C2C12 cells were treated with NAC (3 mM) for 3 h, and ROS levels were quantified by flow cytometry. ROS were stained by H2-DCFDA and green fluorescence was induced. ROS production was inhibited by NAC. (a) Control; (b) NAC (3 mM); y-axis represents cell counts, x-axis represents intensity of green fluorescence. (E) Detection of indicated autophagy-essential protein LC3 by western blot in C2C12 cells with co-treatment of NAC and propofol. Each bar represents a mean ± SD; *, P < 0.05; **, P < 0.01; n = 3 per group; ^##^, p < 0.01 (comparison with cells treated with propofol).

ROS are major metabolites in intracellular signaling cascades that trigger mitochondria-associated events, including apoptosis and autophagy. ROS are generated from various cellular sources, such as NO_X_, mitochondrial respiration, and extracellular stress. Our results showed that propofol significantly stimulated production of intracellular ROS, and this effect was partially mitigated by TUDCA. Indeed, TUDCA alone reduced ROS production, as measured by H2-DCFDA fluorescence (Fig 4A) and flow cytometry (Fig 4B). As exposure to 400 μM propofol did not elicit C2C12 apoptosis, the associated increase in ROS output was likely increased either autophagy or ER stress. Notably, we found that pretreatment for 24 h with 3-MA, an inhibitor of autophagy and class III PI3K, significantly reversed propofol-induced ROS output (Fig 4C). The ROS scavenger N-acetyl cysteine (NAC) was further applied to explore the role of ROS in propofol-induced autophagy. NAC was applied to C2C12 together with 400 μM propofol. ROS levels and LC3 expression were determined (Fig 4D). As expected, ROS levels decreased after NAC treatment. Additionally, the LC3-II/GAPDH ratio and LC3 expression both decreased (Fig 4E). Collectively, the data suggested that propofol treatment is associated with ROS and ER stress, and ROS production enhanced propofol-induced autophagy.

## Discussion

We investigated various phenomena associated with cell autophagy, including ROS production and ER stress, with a view to further elucidate the cellular effects of propofol. We note that, at present, more effective strategies are needed to better manage anesthesia-induced autophagy. Our results indicated that propofol provokes cellular autophagosome accumulation via ER stress and Ca^2+^ homeostasis disruption. ER stress appears to be critical in disruption of Ca^2+^ homeostasis in the presence of propofol. Indeed, propofol-induced autophagy was blocked by TUDCA, an inhibitor of ER stress.

As measured by PCR, immunofluorescence, and western blot, LC3 expression gradually increased after exposure to propofol. We also found that autophagy induction disappeared at 6 h and 24 h. Our results are consistent with those of Kashiwagi *et al* [37], who concluded that muscle autophagy under anesthesia requires two components: anesthesia and muscle denervation. Lack of one or the other does not lead to marked autophagy induction. Denervation and starvation both can induce skeletal muscle autophagy [38]. Our experiment showed that starvation is necessary in propofol-promoted autophagy.

Autophagy is a dynamic process comprising two consecutive stages. The first step is autophagosome formation. The second step is autophagosome clearance, which involves autolysosome formation via autophagosome–lysosome fusion [32, 33]. Evidence suggests that inhibition of autophagy flux may either result in sensitization of cells to stimuli-induced damage or in cell death directly. If autophagosomes are not appropriately cleared, p62 will accumulate within cells [32, 33] which could reflect either induction of autophagic sequestration or reduced autophagosome clearance. Autophagosome clearance is dependent upon the maturation and degradation of autophagosomes, which involves the fusion of autophagosomes with lysosomes to form autolysosomes and the subsequent degradation of luminal substrates [32, 33]. The exact mechanisms underlying autophagosome–lysosome fusion are still unclear and will require further study to clarify. Accordingly, we observed a significant increase in p62 levels after propofol stimulation in C2C12 cells. p62 expression may be transcriptionally increased under certain conditions, further complicating the interpretation of results. For instance, increased p62, or at least transiently increased p62, is sometimes exhibited along with increased autophagic flux [32, 33]. The other explanation is that changes in p62 expression can be cell type- and context-specific.

Our experiments provided an explanation for the mechanism of propofol-induced autophagy. We knew that autophagy due to ER stress was a possible molecular mechanism by which muscle homeostasis is maintained in response to disuse and unloading. Disruption of the homeostasis between cytosolic and organellar Ca^2+^ affects autophagy. Otherwise, disruption of Ca^2+^ homeostasis is related directly to the induction of ER stress [39]. As we observed in our experiments, Ca^2+^ homeostasis recovered after 3 h, releasing the induction of ER stress and autophagy. Explanation of the mechanisms underlying the effects at 6 h and 24 h will require further experiments. Autophagy is a complicated process; in our experiments, we speculate that the effects at 6 h and 24 h could be related to the recovery of Ca^2+^ homeostasis after 3 h, followed by inactivation of ER stress and autophagy.

It is also possible that general anesthesia may modulate autophagy via reorganization of microtubules [40, 41], a critical phase in the formation and maturation of autophagosomes [42]. Our results are consistent with the hypothesis that anesthesia induces autophagy directly, as mitophagy, a form of autophagy, is driven by similar mechanisms [43] and is equally essential in responding to stimuli, as well as in maintaining cellular homeostasis. There is convincing evidence that ER and mitochondrial homeostasis combine to coordinate both stress-induced autophagy and mitophagy to drive the selective engulfment of mitochondria that are in physical contact with the ER. Accordingly, early autophagosome assembly is known to occur at ER-mitochondrial contact sites [44]. In light of this, we consider it important to determine whether propofol promotes mitophagy. Additional research is needed to explore this issue, and to clarify whether propofol impacts mitophagy through IP3R, which physically links the ER and mitochondria.

Our experiments also provided evidence that Ca^2+^ transfer between the ER and mitochondria is a key event in propofol-induced autophagy. However, there is at present no unifying model to explain how Ca^2+^ integrates autophagy pathways [45]. To clarify how ER-mitochondrial Ca^2+^ signaling facilitates propofol-induced autophagy, we will test candidate genes implicated in starvation-induced autophagy. These include *SIGMAR1*, which remarkably interacts with and/or regulates both IP3R and Bip in the ER lumen. *SIGMAR1* also links ER proteostasis to ER-mitochondrial Ca^2+^ signaling and regulates the late phases of autophagosome maturation (autophagic flux). In this study, we have already investigated the expression of IP3R and Beclin-1 in the context of Ca^2+^ transfer [46, 47]. *GRP75* and *MFN2*, which suppress ER-mitochondria Ca^2+^ signaling, are also good candidates for further investigation [17]. Our results are consistent with previous observations [39], as propofol stimulated IP3R and Beclin-1 and disrupted Ca^2+^ homeostasis. These events are clearly related to autophagy upregulation, but for as yet unknown reasons. Whether propofol-induced autophagy require Ca^2+^ should be tested in future experiments using different cell types under various stresses.

Autophagy is essential for cells to maintain homeostasis and survival upon stimulation. The role of propofol-induced autophagy in cell survival remains unclear, and the mechanism is still unknown. Further research is needed to clarify this issue. Some patients with skeletal muscle disorders, including dystrophies and myopathies, also exhibit dysregulated autophagy and are at risk for anesthesia-related complications [48]. Similarly, muscle weakness from immobilization is a risk factor in patients treated in the intensive care unit, where prolonged sedation with ketamine, propofol, or even inhalation of anesthetics occur. Hence, detailed mechanistic investigation is required to fully define the role of immobilization and anesthesia in muscle wasting and atrophy.

## Materials and methods

### Cell culture and drugs

Mouse muscle myoblast C2C12 cells were obtained from the Shanghai Type Culture Collection of China (Shanghai, China), grown to 80% confluence in DMEM supplemented with 10% FBS, and differentiated for two days at 37°C under 5% CO_2_ in DMEM supplemented with 2% horse serum (Solarbio, Beijing, China, S9050-200). When the cells were treated with propofol for the indicated time, we changed the medium from DMEM with 2% horse serum to DMEM without serum, so that autophagy could be induced by starvation. All the experiments were performed under this condition unless stated otherwise. For non-starvation, cells were cultured at 37°C under 5% CO2 in DMEM supplemented with 2% horse serum. Propofol (Sigma-Aldrich, Bogota, Colombia, D126608) was prepared in DMSO and diluted in DMEM to reduce the concentration of DMSO to below 0.01% and thereby minimize its effects. In some experiments, cells were co-treated with 1 mM TUDCA (Aladdin, Shanghai, China, S101371). In others, cells were co-administered with 10 mM 3-MA (Selleckchem, Houston, Texas, USA, S2767) and 3 mM NAC, or 10 μM CQ. All experiments were repeated three times.

### Cell viability

Cell suspensions (7,000 cells/100 μL) were seeded in a 96-well plate, and the cells were grown at 37°C for 48 h, exposed to propofol, and reacted with 10 μL of CCK-8 for 1–2 h, after which the absorbance was measured at 450 nm. Cell viability data are reported as mean ± SD from triplicate experiments.

### Apoptosis

Apoptotic cells were quantified using an annexin V-FITC/PI detection kit (BD Biosciences, USA) and flow cytometry. Briefly, C2C12 cells were treated with 400 μM and 900 μM propofol for 3 h, washed with PBS twice, collected, resuspended in 1× binding buffer, stained for 15 min at room temperature with annexin V and propidium iodide (PI), and analyzed by flow cytometry with CellQuest software (BD Biosciences, Franklin Lakes, NJ, USA).

### RNA extraction and real-time PCR

Total RNA was extracted from C2C12 cells using Trizol reagent (Thermo Fisher Scientific, Waltham, MA, USA). RNA purity and concentration were determined from OD 260/280 reading. Samples were reverse-transcribed using a Go Script Reverse Transcription System (Promega, Madison, WI, USA, A5001) according to the manufacturer’s protocol, and amplified over 40 cycles using FastStart Universal SYBR Green Master mix (Roche, Basel, Switzerland). *BECN1* was amplified with forward primer ATGGAGGGGTCTAAGGCGTC and reverse primer GCCTGGGCTGTGGTAAGTAAT; GAPDH was amplified with forward primer GGCTGCCCAGAACATCAT and reverse primer CGGACACATTGGGGGTAG; LC3 was amplified using forward primer TTGGTCAAGATCATCCGGCG and reverse primer TGGGAGGCGTAGACCATGTA; and p62 was amplified with forward primer GCCAAAGTGTCCATGTTTCA and reverse primer AGGGAACACAGCAAGCT. mRNA levels of target genes were normalized to those of GAPDH, and relative expression was calculated by the 2^-ΔΔCt^ method.

### Western blot

The protein concentration in cell lysates was measured by the Bradford assay (Bio-Rad, Hercules, CA, USA). Samples (30 μg) were separated for 3 h at 80 V on 8–15% SDS-PAGE gels and transferred for 2 h at 100 V to polyvinylidene fluoride membranes, which were then blocked in 4% skim milk for 1 h and probed overnight at 4°C with antibodies (Cell Signaling Technology, Danvers, MA, USA) against CHOP (2895S,1:250), LC3 (4108S,1:500), p62 (5114S,1:500), Beclin-1 (3738S,1:500), AMPKα (2603,1:500), p-AMPKα (4060,1:500), mTOR (2972,1:500), p-mTOR (2971,1:500), Bip (3177P,1:500), IP3R (3763S, 1:500), and GAPDH (2118S1:1000). Membranes were then labeled for 2 h at room temperature with Odyssey secondary antibodies (LI-COR Biosciences, Lincoln, NE, USA), washed three times for 5 minutes each time, and visualized on an Odyssey image analyzer 3.0 (LI-COR Biosciences, Lincoln, NE, USA).

### Immunofluorescence

Propofol-treated cells were fixed with 4% paraformaldehyde for 20 min, treated with 0.3% Triton X-100 for 5 min, washed, and blocked for 30 min at 37°C with 3% bovine serum albumin in Tris-buffered saline containing 0.1% Tween 20. After further washing, samples were incubated overnight with primary antibodies against p62 and LC3 diluted 1:50 in blocking solution. After washing with Tris-buffered saline containing 0.1% Tween 20, fluorescence from secondary antibodies conjugated to horseradish peroxidase was measured on an Operetta high-throughput optical imager (Perkin Elmer, Waltham, MA, USA) using the integrated Columbus image data storage and analysis system.

### Reactive oxygen species

C2C12 cells treated with or without 400 μM propofol were stained with H_2_-DCFDA, an indicator of intracellular ROS. The dye was initially prepared at 10 mM in DMSO and diluted in culture medium to 5 μM. After staining for 30 min, cells were rinsed with PBS three times, incubated for 20 min with Hoechst, and washed with PBS another three times. To confirm the generation of ROS after propofol treatment, H_2-_DCFDA staining was performed. Green fluorescence was analyzed by fluorescence-activated cell sorting and FlowJo software (FlowJo, Ashland, OR, USA). Cells were imaged on an Operetta high-throughput optical imager using the integrated Columbus image data storage and analysis system.

### Rhod-2AM staining

Cells (5,000 cells/well) were seeded in a 96-well plate and incubated at 37°C for 3 h with or without 400 μM propofol, TUDCA, or both. Cells were simultaneously stained with 3 μM Rhod-2AM ester (Biotium, Fremont, CA, USA, 50023) to follow changes in Ca^2+^. Finally, cells were imaged at 30 min, 2 h, and 3 h on an Operetta high-throughput optical imager and analyzed with the integrated Columbus image data storage and analysis system.

### Statistical analysis

Data are reported as mean ± SD with n = 3 (three independent experiments). Groups were compared in SPSS 16.0 (IBM, Armonk, NY, USA) using Student’s two-tailed unpaired t-test or one-way analysis of variance followed by Tukey’s post-hoc test as appropriate. Statistical significance was set at P < 0.05.
